# Influence of Iron and Magnesium on Fouling Properties of Organic Matter Solution in Membrane Process

**DOI:** 10.3390/membranes14070150

**Published:** 2024-07-07

**Authors:** Mohammad T. Alresheedi

**Affiliations:** Department of Civil Engineering, College of Engineering, Qassim University, Buraydah 51452, Saudi Arabia; m.alresheedi@qu.edu.sa

**Keywords:** water treatment, membrane process, iron fouling, magnesium fouling, chemicals removal

## Abstract

Organic matter has been identified as a significant type of foulant in membrane processes for water treatment. Its fouling tendency is highly affected by the presence of ions and inorganics. While the effects of ions addition on organic fouling have been extensively researched in the past, studies on the effect of positively-charged inorganics, such as Fe^2+^ and Mg^2+^, on organic fouling are limited. This study investigates the influence of Fe^2+^ and Mg^2+^ addition on fouling properties of the Suwannee River Organic Matter (SROM) solution in the MF process, with and without Ca^2+^ ions. Results showed that increasing the concentration of Fe^2+^ and Mg^2+^ from 0–5 mM promoted SROM fouling, and resulted in an increased flux decline up to 33% and 58%, respectively. Cake layer resistance became more dominant with the addition of Fe^2+^ and Mg^2+^, and was counted for more than 60% of the fouling. Mg^2+^, however, caused higher internal pore blocking, and facilitated the formation of a less permeable cake layer, compared to Fe^2+^. This was evident in the analysis of the cake layer properties and the visualization of the fouling layer. In all cases, SROM fouling with Fe^2+^ and Mg^2+^ worsened with the addition of Ca^2+^ ions. The results of the study indicated the importance of understanding the interaction between organic matter and Fe^2+^ and Mg^2+^, which would provide useful insights on their fouling mechanism and control.

## 1. Introduction

Membranes have emerged as the treatment process of choice for drinking water production, and in many industrial applications. Membranes are effective in removing a wide range of water contaminants, such as organic substances, inorganics, and microorganisms, and in producing effluents that meet strict water quality standards [1,2]. However, fouling remains a major challenge in membrane processes and their applications in water treatment. Fouling is the deposition of water constituents such as organic matter, inorganics, microorganisms, etc., on the membrane surface or within membrane pores, which results in hindering its performance, reducing flux, and increasing the frequency of membrane cleaning and replacement. Over the past decades, great effort has been made to classify foulants according to their types, fouling mechanism, and control strategies in membrane processes [2,3,4,5,6]. Some common foulant types include organics, inorganics, and colloids. Among these, organic and inorganic substances are the common contributors to membrane fouling during water and wastewater treatment.

Organic matter, such as proteins, humic acids, and polysaccharides, have been identified as essential membrane fouling substances in surface water, seawater, and various types of wastewater. Organic substances generally carry a negative charge on their surfaces at neutral pH, and their fouling tendency is strongly dependent on various factors, such as organic type and concentration, the presence of inorganics and metal cations, pH, and temperature. Among these factors, the presence of inorganics and metal cations has been reported to have a great effect on organic fouling behavior [7,8,9,10].

Inorganic colloidal substances, which are abundant in aquatic environments, including groundwater, surface water, seawater, and wastewater, contribute to membrane fouling. Negatively-charged inorganic colloids, such as silica, can cause many operational issues, including scaling, high-pressure drop, severe flux decline, and deterioration of the membrane separation performance [8,9,11]. Positively-charged inorganic colloids, such as aluminum, have been reported to elevate the severity of membrane fouling due to their higher affinity to adsorb negatively-charged organic substances, which leads to increased organic accumulation on the membrane surface, and flux decline [12,13]. Other studies also demonstrated the effect of inorganic substances on fouling irreversibility and membrane cleaning [14,15,16]. Metal cations such as calcium (Ca^2+^) and sodium (Na^+^) have been reported to have a great effect on organic fouling behavior [10,17,18]. A Ding et al. [17] study reported that increasing Ca^2+^ concentration elevated protein fouling of an MF membrane system. Other studies [7,8,9,10] found that organic fouling can be aggravated by adding Ca^2+^ and Na^+^. A Ca^2+^ and Na^+^ presence can worsen fouling by neutralizing the charge on the organic matter surface and promoting their aggregation, which leads to higher organic matter deposition on the membrane surface, and hence, severe flux loss [10].

Although there are a good amount of studies on the effects of inorganic fouling and Ca^2+^ ions on membrane fouling, the effect of positively-charged inorganic foulants such as Fe^2+^ and Mg^2+^ ions on organic matter fouling problems in membrane processes have been overlooked. Although both Fe^2+^ and Mg^2+^ are ubiquitous in water and present in surface waters, seawater, and various types of wastewater at a low concentration, which can be at a level of several ppm [18,19,20,21], limited effort has been made to understand Fe^2+^ and Mg^2+^‘s effect on membrane processes. For example, studies by Sioutopoulos et al. [22] and Davidkova et al. [23] investigated iron fouling in RO and NF membranes, and found that the presence of Fe^2+^ colloids enhanced fouling of the RO and NF membrane, and resulted in great flux decline. Moreover, in well-oxygenated waters, Fe^2+^ can be easily converted to Fe^3+^, which can form insoluble colloids. These colloids can cause fouling problems if the membrane is not proceeded by the pretreatment stage; furthermore, if they are not removed, these insoluble colloids can build up on the membrane over time and decrease its operational life [23]. A Xin et al. [24] study showed different results, in which reduced fouling of alginate at a high iron concentration was observed. However, the level of fouling was dependent on the concentration of alginate in the solution. Some other studies [17,25,26,27] investigated the effect of Mg^2+^ on fouling in membrane processes; however, different outcomes were reported. For example, a study by Wang et al. [26] reported that the addition of Mg^2+^ ions aggravated polysaccharide fouling of the UF process. On the contrary, a study by Zou et al. [27] reported mitigated protein fouling with the addition of Mg^2+^ ions. Previous studies also pointed out that Ca^2+^ ions influence fouling of organic matter and enhance the formation of the compact fouling layer and higher surface roughness [23,25]. The reported effect of Ca^2+^ on fouling differs from Fe^2+^ and Mg^2+^. For example, a study by Wang et al. [25] showed that Ca^2+^ has a higher charge neutralization capacity and more favorable binding ability compared to Mg^2+^. Other studies pointed out that the effect of Fe^2+^ and Mg^2+^ on the fouling behavior of sodium alginate was different from that of Ca^2+^ due to the large hydration radius of Fe^2+^ and Mg^2+^ [21,24], underlining the need for more research in this area.

While the previous limited studies have pointed out the effect of inorganics on membrane fouling, more research is needed to understand the effect of positively-charged inorganics such as Ca^2+^, Fe^2+^, and Mg^2+^ on organic matter fouling behavior. Since the aforementioned positively-charged inorganics and negatively-charged organic matter can co-exist in various types of water, their interactions and combined effect on membrane fouling needs further exploration. Therefore, this study aims to investigate the influence of Fe^2+^ and Mg^2+^ on fouling properties of organic matter solution in membrane processes. Suwannee River Organic Matter (SROM) was used as the model of organic foulant. The fouling layer formed from the tested solutions with various SROM and Fe^2+^ and Mg^2+^ concentrations, with and without Ca^2+^ ions, was examined under constant pressure dead-end microfiltration (MF). The performance of the MF membrane, with regard to fouling mechanism, was assessed through permeability, fouling resistances, zeta potential, fouling layer properties, and analysis of the formed fouling layer. This study provides insights into Fe^2+^ and Mg^2+^ effect on organic fouling in a membrane filtration process, which would be useful for fouling control and membrane operation optimization.

## 2. Materials and Methods

### 2.1. Feed Solution Preparation and Characterization

The feed solutions were prepared using SROM (International Humic Substances Society) as the model of organic matter. Then, 20 mg/L of SROM was prepared by dissolving the powdered SROM in pure Milli-Q water (Millipore, Burlington, MA, USA) and stirring for at least 24 h prior to experiment at room temperature. Stock solutions of Fe^2+^ (FeCl_3_⋅6H_2_O; Sigma, Burlington, MA, USA) and Mg (MgCl_2_⋅6H_2_O; Sigma, Burlington, MA, USA) were prepared. Fe^2+^ and Mg^2+^ were added by pipetting different concentrations of 0, 0.5, 1, 2, 5, and 10 mM into the prepared SROM solution. The Fe^2+^ and Mg^2+^ concentrations have been selected based on previous studies [24,26]. For the case of studying the effect of Ca^2+^, 1 mM of CaCl_2_ was added to the SROM solution, followed by the addition of the desired amount of Fe^2+^ and Mg^2+^ under continuous mixing. The solution’s pH was controlled around pH 7.2 using NaOH prior to filtration.

The zeta potential and particle size of SROM solutions with different Fe^2+^ and Mg^2+^ concentrations, with and without Ca^2+^ ions, were measured using a Malvern Zetasizer Nano (Malvern Instruments, Malvern, UK). In brief, SROM solution (or in some cases, SROM and Ca^2+^ solution) was stirred overnight. The zeta potential and size of the SROM solution were measured using the Malvern Zetasizer Nano before and after Fe^2+^ or Mg^2+^ addition. pH values of the sample were measured and adjusted as required. Each measurement was repeated at least 3 times, and average values were reported.

### 2.2. Filtration Tests

Fouling experiments were performed on a laboratory dead-end filtration setup. The setup included a stirred filtration cell reservoir (Merck, Burlington, MA, USA), a nitrogen tank to provide the required filtration pressure, and a filtrate beaker placed on a digital balance (OHAUS, Parsippany, NJ, USA). A flat sheet-regenerated cellulose MF membrane (Millipore Co., USA.) with a pore size of 0.1 µm was used. The MF membrane was cleaned with Milli-Q water for at least 24 h before the experiment. Milli-Q water was then passed through the membrane at a set pressure of 1 bar to stabilize the permeate flux, and to determine the intrinsic membrane resistance (R_m_). For fouling experiments, the SROM solution with the desired Fe^2+^ and Mg^2+^ concentration, with and without Ca^2+^ ions, was then filtered under a filtration pressure of 1 bar, which was provided by the nitrogen gas. Each fouling experiment was conducted for 180 min (3 h.) at room temperature (22 °C). Filtrate water was then collected in a beaker on a digital balance. The volume of the filtrate water was recorded every 20 s by the balance, which was connected to a personal computer. Fouled membranes were then removed from the filtration cell for analysis. New fresh membrane was used in each experiment.

### 2.3. Fouling Mechanism Analysis

#### 2.3.1. Estimation of Fouling Resistances

Membrane flux (J) during filtration can be defined as the relationship between applied pressure (Δp), total filtration resistance (R_t_), and water viscosity (µ) (Equation (1)). R_t_ during the filtration of SROM with Fe^2+^ and Mg^2+^, with and without, Ca^2+^ ions was determined using the resistance in series model [28]. R_t_ (Equation (2)) was divided into intrinsic (clean) membrane resistance (R_m_); pore blocking resistance (R_pp_); and cake layer resistance (R_cl_).

(1) $$J=\frac{\Delta P}{\mu Rt}$$

R_t_ = R_m_ + R_cl_ + R_pp_ (2)

R_m_ was estimated before and between experiments using Equation (1), by filtering Milli-Q water through a clean filter at constant pressure at room temperature, and then recording the J for at least 60 min. R_t_ was determined using flux and pressure data during fouling experiments. After the filtration period (i.e., 180 min), fouled membranes were gently cleaned and rinsed with Milli-Q water to remove the cake layer. Milli-Q water was then filtered to determine the R_pp_. R_cl_ was then back-calculated using the difference between the R_t_, R_pp_, and R_m_, as shown in Equation (2). Where J is in (m/s); R_t_, R_m_, R_cl_, and R_pp_ are in (m^−1^); ΔP is in (Pa); and µ is in (Pa s). The changes in flux and pressure data with time were used to calculate the fouling resistances.

#### 2.3.2. Fouling Layer Properties

A specific cake resistance α_c_ (m/gC) and cake compressibility index (n) analysis of the foulant layer was conducted to evaluate the changes in properties of the membrane surface structure and foulant layer at the tested conditions [29,30]. α_c_ is a function of the cake layer resistance (R_c_), particle concentration, C_b_ (kg/m^3^), and the filtered water volume, V_s_ (m^3^/m^2^), as shown in Equation (3).

(3) $$\alpha c=\frac{Rc}{\begin{array}{c} Cb\;Vs \end{array}}$$

The α*_c_* increases according to a power law (Equation (4)), in which a function of the applied pressure (ΔP) and the properties of particles form the cake (α_0_) [29,30], as shown in Equation (4). The n was then determined as follows, by plotting the α_c_ and ΔP:

α_c_ = α_0_ × ΔP^n^ (4)

#### 2.3.3. SEM Imaging

To gain further insights on the fouling layer formed by SROM and Fe^2+^ and Mg, with and without Ca ions, scanning electron microscopy (SEM) analysis was conducted on the fouled membranes. SEM images were taken using the Tescan Vega-II XMU equipment (Warrendale, PA, USA). Fouled membranes were placed overnight to dry at room temperature prior to SEM analysis.

## 3. Results and Discussion

### 3.1. Characterization of SROM Feed Solution with Different Fe^2+^ and Mg^2+^ Concentrations

#### 3.1.1. Zeta Potential of Feed Solutions

**Figure 1:**
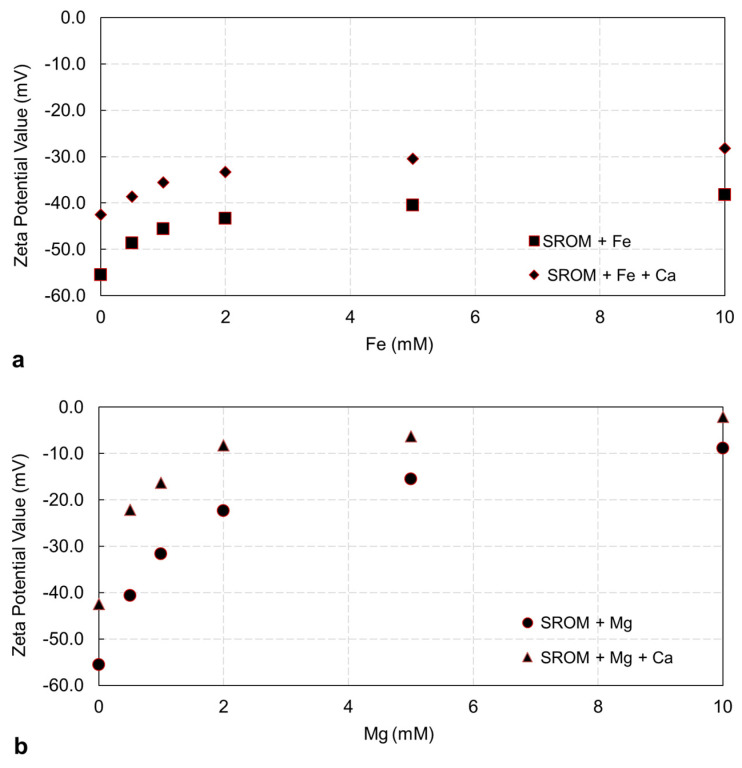
Zeta potentials of SROM solution with different Fe^2+^ and Mg^2+^ concentrations, with and without Ca^2+^ addition. (**a**) SROM + Fe^2+^; (**b**) SROM + Mg^2+^.

Figure 1 presents the zeta potentials of SROM solutions with different Fe^2+^ and Mg^2+^ concentrations, with and without the Ca^2+^ addition. It can be seen that, for SROM solution alone, the zeta potential value was negative, −55.5 mV, indicating that SROM particles were stable and had less tendencies of aggregation. With the addition of Fe^2+^ to SROM (Figure 1a), the zeta potentials of the mixture solution became less negative (i.e., less stable), and values increased moderately from increasing the Fe^2+^ concentration from 0.5–10 mM, which reached –38.2 mV at 20 mg/L of SROM and 10 mM of Fe^2+^. The addition of Mg^2+^ to the SROM solution (Figure 1b), however, showed a significant increase in the zeta potential values; that is, the addition of 0.5 mM of Mg^2+^ resulted in a zeta potential of −40.6 mM, and values increased from increasing the Mg^2+^ concentration from 0.5–10 mM, which reached –8.8 mV at 20 mg/L of SROM and 10 mM of Mg^2+^. These results indicate that although both Fe^2+^ and Mg^2+^ reduced the negative charge on the SROM, the zeta potentials of the SROM solution were dependent on the level of the Fe^2+^ and Mg^2+^ addition; that is, the SROM net charge was neutralized with the increase of the Fe^2+^ and Mg^2+^ concentration. Mg^2+^ ions have a more pronounced effect on the zeta potential of SROM compared to Fe^2+^, resulting in less stable SROM. A study by Xin et al. [23] reported that there was little effect of the Fe^2+^ addition on the zeta potential of alginate solution. This could be attributed to the different model of organic matter used, and the low concentration range of Fe^2+^ used in that study. In another study by Davidkova et al. [22], the addition of organic to iron oxide solution resulted in a surface charge reversal from positive to negative, which led to higher fouling and flux decline. The MF membrane surface used in this study had a negative zeta potential; thus, it is expected that electrostatic repulsion between the MF membrane surface and the SROM will be reduced from increasing the Fe^2+^ and Mg^2+^ concentration, suggesting a higher tendency for SROM to deposit on the membrane surface, and thus, higher fouling.

Figure 1 also shows the effect of the Ca^2+^ addition on the zeta potentials of the SROM solution. The addition of 1 mM Ca^2+^ increased the zeta potentials’ values by approximately 30% for the tested SROM solution. Similar to the case without Ca^2+^ (as shown in Figure 1a,b), a further Fe^2+^ and Mg^2+^ addition had a pronounced effect on the zeta potential values, resulting in less stable particles. Previous studies [23,31,32,33] have reported that cations can neutralize the surface charge of organic matter and promote their fouling tendency, which is aligned with the results here.

#### 3.1.2. Particle Size Analysis of Feed Solutions

**Figure 2:**
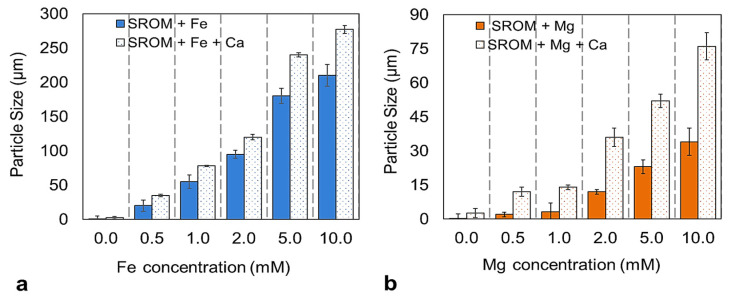
Particle size of the SROM solution with different Fe^2+^ and Mg^2+^ concentrations, with and without Ca^2+^ addition. (**a**) SROM + Fe^2+^; (**b**) SROM + Mg^2+^.

Figure 2 illustrates the effect of the Fe^2+^ and Mg^2+^ addition on the particle size of SROM, with and without the Ca^2+^ addition. As shown in Figure 2, the particle size of SROM alone increased from 0.22–20.6 µm as a result of increasing Fe^2+^ concentration from 0–0.5 mM, and continued to increase to 210.3 µm when the Fe^2+^ concentration increased to 10 mM. Unlike Fe^2+^, the addition of up to 1 mM of Mg^2+^ resulted in almost no observed particle size growth (i.e., SROM size only increased from 0.22–3.1 µm at 0 and 1 mM of Mg^2+^). However, from increasing Mg^2+^ concentration to 2 mM, a significant increase in the size of the SROM particles was observed, which reached 14.8 µm and continued to increase to 34.2 µm when the Mg^2+^ concentration increased to 10 mM. With the addition of 1 mM of Ca^2+^, and in the absence of Fe^2+^ and Mg^2+^, the size of SROM particles was increased to 2.6 µm, which was almost similar to that of 1 mM of Mg^2+^ alone. The addition of Ca^2+^ to the SROM solution at 0.5 mM of Fe^2+^ and Mg^2+^ formed large particles and continued to increase to 277.1 and 76.3 µm when the Fe^2+^ and Mg^2+^ concentration increased to 10 mM, respectively. It was previously reported that both zeta potential and particle size play an important role in membrane fouling and performance [23,34,35]. While zeta potential determines the tendency of particles to aggregate and grow, particle size determines how particles accumulate on the membrane surface (i.e., internally or externally). Smaller particles tend to accumulate within the membrane pores or block the pores completely, whereas large particles accumulate externally on the membrane surface and form a cake layer [13,36]. According to the zeta potential values in Figure 1 and the particle size change in Figure 2, it can be suggested that with the addition of Fe^2+^ and Mg^2+^ at different concentrations to the SROM, with and without Ca^2+^ ions, the SROM became less stable, with larger particle sizes compared to the SROM alone. These two characteristics are expected to influence the SROM filtration performance and fouling behavior. It is worth pointing out that in this study, all testing was conducted at room temperature (22 °C); thus, the effect of viscosity on the adhesion behavior of the pollutants was negligible. Additionally, Xin et al.’s [24] study reported minimal changes in solution viscosity with changing alginate and iron concentration.

### 3.2. Effect of Fe^2+^ and Mg^2+^ Concentration on the Filtration Performance of SROM

**Figure 3:**
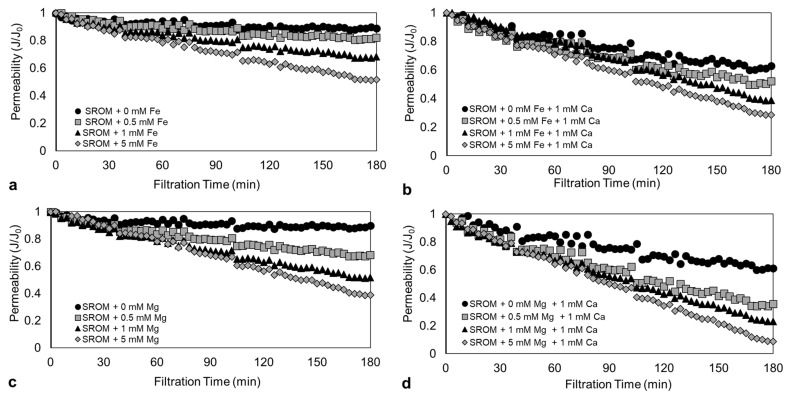
SROM filtration profiles at different Fe^2+^ and Mg^2+^ concentrations. (**a**) SROM + Fe^2+^; (**b**) SROM + Fe^2+^ + Ca^2+^; (**c**) SROM + Mg^2+^; (**d**) SROM + Mg^2+^ + Ca^2+^.

Figure 3a–d present the membrane permeability profiles during filtration of SROM with Fe^2+^ and Mg^2+^, with and without Ca^2+^. The results of Fe^2+^ and Mg^2+^ concentrations of 0, 0.5, 1, and 5 mM were presented here as examples. As can be seen in Figure 3a, the filtration of SROM alone caused an immediate decline in membrane permeability within the first 30–60 min, and reached a ratio of 0.87, which remained unchanged for the remaining filtration period. The addition of 0.5 mM of Fe^2+^ to the SROM solution resulted in a moderate decline in permeability, which reached a ratio of 0.81 (6% decline) at the end of the filtration period (i.e., 180 min). Furthermore, increasing the Fe^2+^ concentration to 1 and 5 mM resulted in a 20% and 33% decline in permeability, which reached a ratio of 0.68 and 0.52, respectively. The decline in membrane permeability with increasing Fe^2+^ concentration could be attributed to the reduced negative charge of the surface of SROM with the Fe^2+^ addition, which may have enhanced fouling. This is aligned with other studies that reported increased fouling with increasing positively-charged ions in water [22,23].

Unlike Fe^2+^, the addition of 0.5 mM of Mg^2+^ to the SROM (Figure 3c) resulted in a much faster and sharper decline in permeability, which resulted in a final ratio of 0.66 (24% decline). Moreover, increasing the Mg^2+^ concentration to 1 and 5 mM decreased permeability by 45% and 58%, which reached a value of 0.47 and 0.36. The fouling trend for SROM in the presence of Mg^2+^ is similar to that for Fe^2+^; however, the level of permeability decline differs. As shown in the zeta potential results, the Mg^2^ addition significantly reduced the negative surface charge of the SROM, which promoted its deposition on the membrane surface and within its pores. The particle size of the Mg^2+^ was smaller than that of Fe^2+^, which may cause an internal pore fouling, followed by the formation of a less permeable cake layer. This resulted in the sharp decline in permeability compared to that of Fe^2+^. The effect of particle surface charge and size on fouling was also reported in other studies [13,22,36].

Figure 3b,d show the effect of the Ca^2+^ addition on the filtration profile of the SROM solution with Fe^2+^ and Mg^2+^. It showed that the addition of 1 mM Ca^2+^ to SROM alone (i.e., in the absence of Fe^2+^ and Mg^2+^) enhanced fouling and permeability decline by approximately 28%, which reached a final ratio of 0.63. The fouling behavior of the SROM with Ca^2+^ was expected, as Ca^2+^ ions can promote the aggregation of organic particles through surface neutralization [23,37]. Similar to the case without Ca^2+^, further Fe^2+^ and Mg^2+^ additions had a pronounced effect on the SROM fouling behavior and permeability decline; that is, the SROM fouling with Fe^2+^ and Mg^2+^ worsened in the presence of Ca^2+^ ions (as shown in Figure 3b,d). Membrane permeability in the presence of Ca^2+^ ions, declined by 17–55% and 44–78% from increasing the Fe^2+^ and Mg^2+^ concentration from 0.5–5 mM, respectively. Therefore, analysis of the SROM fouling resistance and foulant layer with Fe^2+^ and Mg^2+^, with and without Ca^2+^ ions, was needed to understand the fouling mechanism.

### 3.3. Analysis of SROM Fouling with Different Fe^2+^ and Mg^2+^ Concentrations

#### 3.3.1. Cake Layer and Pore Blocking Resistances

**Figure 4:**
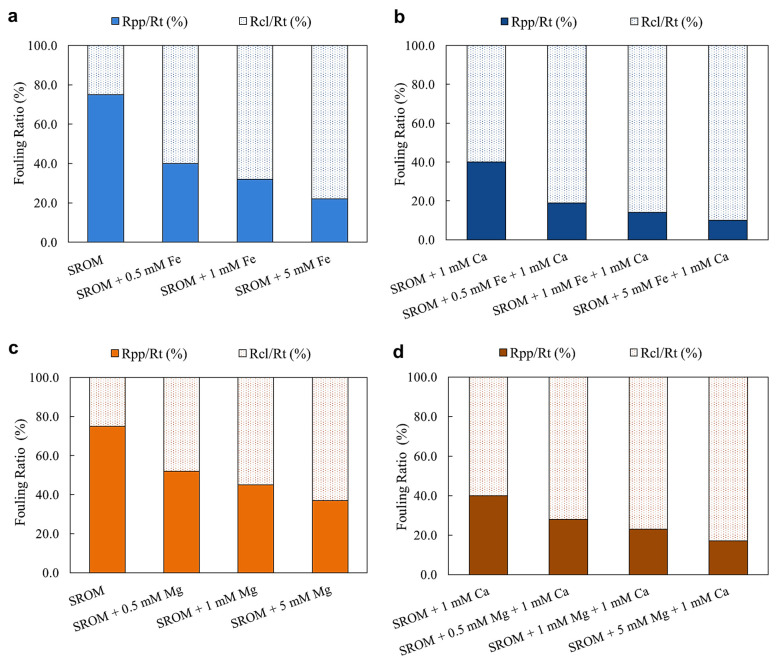
Fouling resistances of SROM during filtration with different concentrations of Fe^2+^ and Mg^2+^. (**a**) SROM + Fe^2+^; (**b**) SROM + Fe^2+^ + Ca^2+^; (**c**) SROM + Mg^2+^; (**d**) SROM + Mg^2+^ +Ca^2+^.

Figure 4a–d illustrate the fouling resistance ratios due to pore blocking (R_pp_) and the cake layer (R_cl_) during filtration of SROM with different Fe^2+^ and Mg^2+^ concentrations (using 0, 0.5, 1, and 5 mM as examples), with and without Ca^2+^ ions.

Results showed that for the filtration of SROM only, 75% of fouling was due to pore blocking, whereas the remaining 25% was due to cake filtration (Figure 4a,c). However, with the addition of 0.5 mM of Fe^2+^ or Mg^2+^, the cake fouling resistance ratio increased to 60% and 48%, respectively. As a result of increasing the Fe^2+^ and Mg^2+^ concentration from 0.5 mM to 5 mM, cake resistance became more dominant, and was responsible for more than 75% and 60% of the fouling for Fe^2+^ and Mg^2+^, respectively. Although the fouling resistance trends of Fe^2+^ and Mg^2+^ were similar, Mg^2+^ caused a higher degree of pore blocking compared to Fe^2+^, which explains the higher permeability decline caused by Mg^2+^. These results are aligned with the permeability decline results presented in Figure 3.

Figure 4b,d show the effect of the Ca^2+^ addition on the fouling resistances of the SROM solution with Fe^2+^ and Mg^2+^. Results showed that the addition of 1 mM Ca^2+^ to SROM alone (i.e., in the absence of Fe^2+^ and Mg^2+^) enhanced the cake resistance ratio by almost 35%, compared to SROM alone. The fouling behavior of SROM with Ca^2+^ was expected, considering that Ca^2+^ ions aggregated with SROM and increased their particle size, which may have resulted in higher cake layer formation. This was evident in the particle size analysis, and in the filtration profiles of SROM (Figure 2 and Figure 3). Additionally, in the presence of Ca^2+^, further Fe^2+^ and Mg^2+^ additions had a pronounced effect on the SROM fouling resistances; that is, the SROM fouling due to cake filtration increased with Fe^2+^ and Mg^2+^ with Ca^2+^ ions (as shown in Figure 4b,d). The fouling ratio due to cake formation in the presence of Ca^2+^ ions, increased by 35–50% and 20–38% from increasing Fe^2+^ and Mg^2+^ concentrations from 0.5–5 mM, respectively. The changes in SROM fouling resistance with Fe^2+^ and Mg^2+^ in the presence of Ca^2+^ indicated the importance of altering membrane backwash and cleaning procedures based on feed water composition to control fouling and improve permeability.

#### 3.3.2. Fouling Layer Properties

Table 1 and Table 2 present the fouling layer properties of the model foulants used in this study. The specific cake resistance (α_c_) and the cake compressibility index (n) were determined using Equations (3) and (4).

The α_c_ caused by SROM alone was 1.56 × 10^3^, with a low n value of 0.28. This indicates that the fouling layer formed by SROM was more porous, with an open structure, which agrees with the slower decline in membrane permeability (Figure 3). Increasing the concentration of Fe^2+^ in the solution from 0.5–5 mM resulted in an increase in the cake resistance, and a more compressible cake layer. Similarly, increasing the concentration of Mg^2+^ in the solution from 0.5–5 mM resulted in a significant increase in the cake resistance, and a highly compressible cake layer. The addition of Mg^2+^ to SROM had a relatively higher effect on the cake layer properties compared to Fe^2+^. This could be related to the small particle size of Mg^2+^ compared to Fe^2+^, which may cause internal pore blocking and facilitate the formation of a less permeable cake layer.

Table 1 and Table 2 also show the effect of the Ca^2+^ addition on the cake layer properties of the SROM solution with Fe^2+^ and Mg^2+^. The addition of 1 mM Ca^2+^ to SROM alone (i.e., in the absence of Fe^2+^ and Mg^2+^) increased α_c_ and n values almost 2-fold, compared to SROM alone. Other studies [13,23,38,39,40] have reported the Ca^2+^ effect on the cake layer structure and fouling irreversibility, which is aligned with the results of this study. Additionally, in the presence of Ca^2+^, further Fe^2+^ and Mg^2+^ additions had a pronounced effect on the SROM fouling layer properties; that is, the α_c_ of SROM increased with Fe^2+^ and Mg^2+^ in the presence of Ca^2+^ ions (as shown in Table 1 and Table 2). n value increased 1.5–2.5-fold from increasing the Fe^2+^ and Mg^2+^ concentration from 0.5–5 mM, with and without Ca^2+^ ions.

#### 3.3.3. SEM Analysis of Fouled Membranes

**Figure 5:**
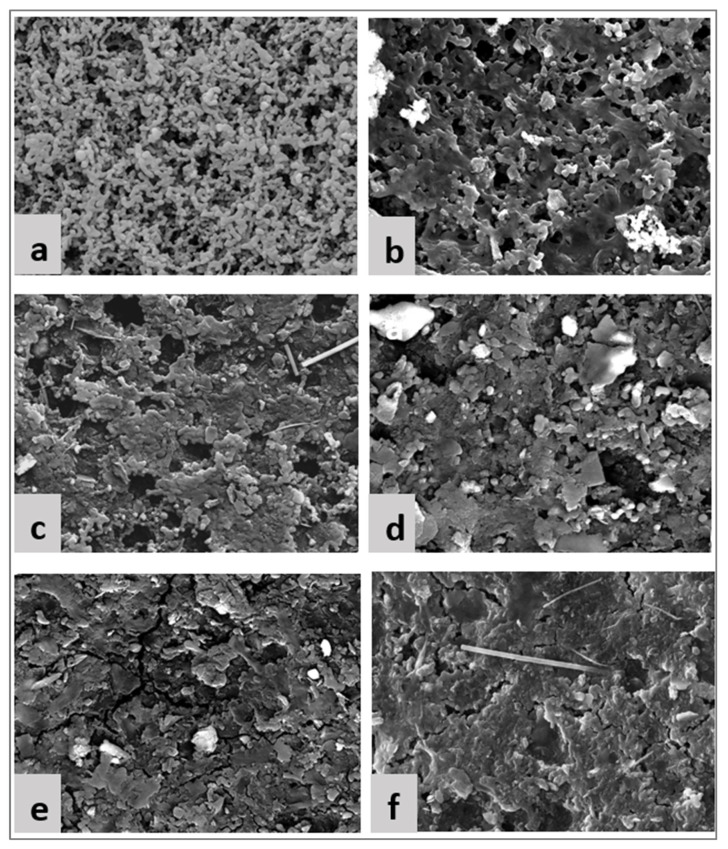
SEM images of membrane surface. (**a**) Clean membrane; (**b**) SROM; (**c**) SROM + 5 mM Fe^2+^; (**d**) SROM + 5 mM Fe^2+^ + 1 mM Ca^2+^; (**e**) SROM + 5 mM Mg^2+^; (**f**) SROM + 5 mM Mg^2+^ + 1 mM Ca^2+^.

SEM image analyses were performed to gain a deep understanding of the fouling layer formed on the membrane surface. Figure 5 shows a clean membrane surface compared to the fouled surface (using 5 mM of Fe^2+^ and Mg^2+^ as examples). In the case of filtration of SROM alone (Figure 5b), the fouling layer formed was thin and loose with noticeable open pores. The SROM alone, due to its small size, formed a thin cake layer with an open structure, which indicates that SROM may cause internal pore fouling. This is in agreement with the filtration resistance analysis (refer to Figure 4), which showed a higher percentage of pore blocking in the case of SROM filtration alone. In the case of filtration of SROM with Fe^2+^ (Figure 4c), a noticeable thick and compact gel layer was formed, wherein decreased open pores were observed, compared to that of SROM alone. This indicated that with the addition of Fe^2+^ to SROM, fouling transitioned from pore blocking to cake formation, which was consistent with the fouling resistance’s analysis in Figure 4. Moreover, this was reflected in the increased decline in membrane permeability with the Fe^2+^ addition (Figure 3). Similarly, the addition of Mg^2+^ affected the fouling layer structure of SROM, as the fouling layer became extraordinary different, with a much denser structure and almost no visible open pores on the surface (Figure 5e). This was consistent with the results of fouling resistance and permeability decline, which also showed the role of Mg^2+^ in facilitating a combination of pore blocking and cake layer formation. The addition of Ca^2+^ ions worsens the fouling layer of SROM with Fe^2+^ and Mg^2+^ (as shown in Figure 5d,f), which demonstrates the profound effect of Ca^2+^ on fouling formation and membrane permeability.

## 4. Conclusions

This study investigated the influence of Fe^2+^ and Mg^2+^ additions on fouling properties of the SROM solution in the MF process, with and without Ca^2+^ ions. Key findings were as follows:

- Fe^2+^ and Mg^2+^ ions have a pronounced effect of the zeta potential of SROM, which resulted in less stable particles.
- Increasing the concentration of Fe^2+^ and Mg^2+^ from 0–5 mM promoted SROM fouling, and resulted in an increased flux decline of up to 33% and 58%, respectively.
- For SROM only, 75% of fouling was due to pore blocking, whereas the remaining 25% was due to cake filtration. The cake layer resistance became more dominant with the addition of Fe^2+^ and Mg^2+^, and was responsible for more than 60% of the fouling. Mg^2+^, however, caused higher internal pore blocking and facilitated the formation of a less permeable cake layer compared to Fe^2+^. This was evident in the analysis of the cake layer properties and the visualization of the fouling layer.
- In all cases, SROM fouling with Fe^2+^ and Mg^2+^ worsened in the presence of Ca^2+^ ions due to charge neutralization and aggregation of SROM; hence, it has a high fouling tendency.
- The results of the study indicate the importance of understanding the interaction between organic matter and Fe^2+^ and Mg^2+^, which would provide useful insights on their fouling mechanism and control. Future studies on the fouling behavior of other positively-charged ions and heavy metals from different water and wastewater sources in membrane processes are recommended.

## Figures and Tables

**Table 1:** SROM fouling layer properties with Fe^2+^, with and without Ca^2+^.

Foulant	Specific Cake Resistance α_c_ (m/gC), (×10^3^)	Cake Compressibility Index (n)
SROM + 0 mM Fe^2+^	1.56	0.28
SROM + 0.5 mM Fe^2+^	2.22	0.41
SROM + 1 mM Fe^2+^	4.14	0.55
SROM + 5 mM Fe^2+^	5.36	0.68
SROM + 0 mM Fe^2+^ + 1 mM Ca^2+^	3.52	0.44
SROM + 0.5 mM Fe^2+^ + 1 mM Ca^2+^	6.54	0.63
SROM + 1 mM Fe^2+^ + 1 mM Ca^2+^	8.21	0.76
SROM + 5 mM Fe^2+^ + 1 mM Ca^2+^	9.53	0.88

**Table 2:** SROM fouling layer properties with Mg^2+^, with and without Ca^2+^.

Foulant	Specific Cake Resistance α_c_ (m/gC), (×10^3^)	Cake Compressibility Index (n)
SROM + 0 mM Mg^2+^	1.56	0.28
SROM + 0.5 mM Mg^2+^	3.22	0.46
SROM + 1 mM Mg^2+^	5.44	0.65
SROM + 5 mM Mg^2+^	6.89	0.72
SROM + 0 mM Mg^2+^+ 1 mM Ca^2+^	3.52	0.44
SROM + 0.5 mM Mg^2+^ + 1 mM Ca^2+^	7.88	0.71
SROM + 1 mM Mg^2+^ + 1 mM Ca^2+^	9.13	0.85
SROM + 5 mM Mg^2+^ + 1 mM Ca^2+^	9.96	0.94

## Data Availability

All required data are included in the main manuscript.
